# Ethnomedicinal Practices for Treating Liver Disorders of Local Communities in the Southern Regions of Korea

**DOI:** 10.1155/2013/869176

**Published:** 2013-09-08

**Authors:** Hyun Kim, Mi-Jang Song

**Affiliations:** School of Alternative Medicine and Health Science, Jeonju University, 303 Cheonjam-ro, Wansan-gu, Jeonju 560-759, Republic of Korea

## Abstract

This study aims to analyze and record ethnomedicinal practices for treating liver disorders of residents living in local communities in the southern regions of Korea. Data was collected using participant observations and in-depth interviews, as the informants also become investigators themselves through attending informal meetings, open and group discussions, and overt observations with semistructured questionnaires. In this study, ethnomedicinal practices for liver ailments were recorded by 1,543 informants (362 men, 1,181 women) at 160 sites. The kinds of liver disorders treated by ethnomedicinal practices were liver cancer, liver cirrhosis, jaundice, hepatitis, fatigue recovery, hangovers, and liver-related ailments. The category with the highest degree of consensus from the informants was jaundice (0.95), and the lowest degree of consensus was for liver cancer and liver cirrhosis (0.61). According to fidelity levels, 28 species resulted in fidelity levels of 100%. The internetwork analysis was first applied for the interpretation of ethnomedicinal knowledge of a community, although it has been strictly used until now for social science in the analysis of social trends and phenomena through the interrelationship of specific components.

## 1. Introduction

After the agreement of the Nagoya Protocol, a stronger interest for sharing the ethnomedicinal knowledge of genetic resources and their benefits to the world has occurred [1]. The ethnomedicinal practices hold significant value among the traditional knowledge of the local communities [2]. Also, ethnomedicinal practices hold an important position in caring for health issues in developing countries [3, 4]. Particularly, the ethnomedicinal practices of regions known for traditional medicine include China, Tibet, and India, nations which have actively utilized these treatments for health concerns. 

The ethnomedicinal practices of Korea have been continuously successful for over 3,000 years. Korean ethnomedicinal practices contain not only medicinal practice, which include single-medicine proscription, herb fomentation, herb fumigation, and herb ablution, but also nonmedicinal practices like acupuncture, moxibustion, Korean traditional therapeutic touch, Korean traditional saunas, and Korean traditional divination practice. These days, Korean ethnomedicinal practices have been restricted to oral transmission because government policy, after the Korean War, centered on conventional medicine, and the Japanese imperialism, which Korea endured from 1919 to 1945, attempted to annihilate its culture [5].

A scientific investigation of liver disease, the first of its kind, was conducted on indigenous communities in Uttarakhand (India) and was related to jaundice research [6]. In Korea, studies on disease within local communities have been conducted on three separate occasions by the authors: respiratory diseases [7], diseases related to digestion [8], and pain relief [9]; however, this research is the first one to focus strictly on liver disorders.

Our research on liver disorders contains an important meaning to first record and analyze the ethnomedicinal practices of local communities in East Asia, including Korea. Additionally, our research newly included the quantitative analysis method known as internetwork analysis (INA), which has allowed more information to be attained in regard to ethnomedicinal knowledge.

Generally, quantitative analysis for ethnomedicinal knowledge of local communities has solely relied on the consensus of the informants and the recorded fidelity levels; therefore, a need exists to utilize the internetwork analysis method to consider the traditional ethnographical properties.

Particularly, a deeper analysis of ethnomedicinal practices in treating specific diseases within the local communities is necessary for obtaining more specific details regarding the internetwork of the components within ethnomedicinal knowledge.

Our research suggests that the applications gained from utilizing the internetwork analysis (INA) for ethnomedicinal practices on liver disorders within communities in Korea will result in further research incorporating INA.

## 2. The Research Area and Method

### 2.1. Natural and Social Environment of Research Area

**Figure 1:**
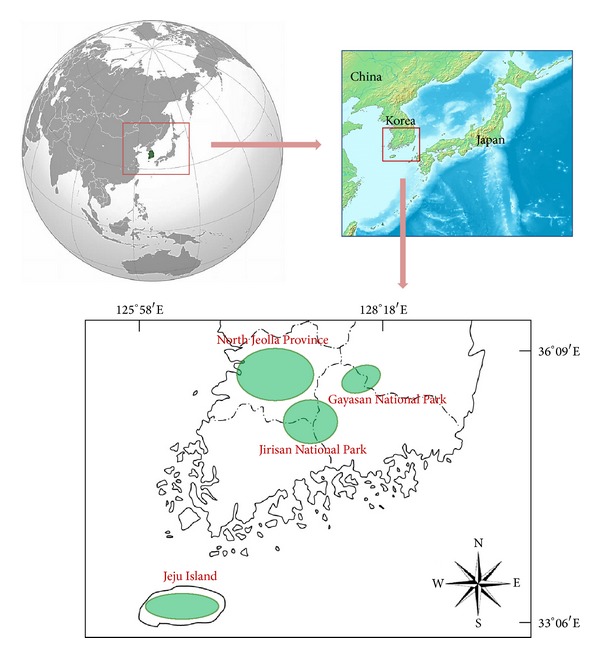
Investigation sites.

The study area consists of the southern region of the Korean peninsula and its many islands, which lie between 33°06′N to 36°09′N latitude and 125°58′E to 128°18′E (Figure 1). The total population in 2012 of the study area was 5,914,270. The area measures approximately 485 km^2^ and includes three provinces, 15 cities, and 27 counties in its administrative district [10]. The annual precipitation is around 1,000~1,850 mm in which the coastal area generally receives more rainfall than the inland regions. The annual average temperature of the inland regions is 13.8°C while Jeju Island records 16.2°C [11].

### 2.2. Research Methods

Field investigations were conducted from March 2009 to November 2012. Proper data was collected using participant observations and in-depth interviews, as the informants also become investigators themselves through attending informal meetings, open and group discussions, and overt observations with semistructured questionnaires [12, 13]. 

The content of the semi-structured questionnaires was composed of diverse information regarding medicinal species used to treat liver disorders, including local names, used parts, ailments, methods of preparation, manufacturing and administration, dosage, and the usable duration regarding each curable formula [13–15].

All specimens were collected during their flowering or fruiting seasons and were organized utilizing the normal specimen manufacturing method [15, 16]. The voucher specimens were deposited for preservation in the herbarium of Jeonju University. The precise identification of species mentioned by the informants was performed in accordance with Lee [17], Lee [18], Ahn [19], Lee [20], and Park [21]. Scientific names were confirmed by the National Knowledge and Information System for Biological Species [22] of Korea.

### 2.3. Quantitative Analysis 

#### 2.3.1. Informant Consensus Factor (ICF)

The ICF was used to analyze the agreement degree of the informants' knowledge about each category of ailments [23, 24]. The ICF was calculated using the following formula:

(1) $$\begin{array}{c} \text{ICF}=\frac{\left(n_{\text{ur}}-n_{t}\right)}{\left(n_{\text{ur}}-1\right)}, \end{array}$$

where *n*_ur_ is the number of use reports of informants for a particular liver disorder and *n*_*t*_ is the number of species used by all informants for a particular liver disorder.

#### 2.3.2. Fidelity Level (FL)

The FL was employed to determine the most important species used for treating certain liver disorders by the local practitioners and the elderly people living in the study area [13, 16, 25]. The FL was calculated using the following formula:

(2) $$\begin{array}{c} \text{FL}\left(\%\right)=N_{p}\times \frac{100}{N}, \end{array}$$

where *N*_*p*_ is the number of informants that mentioned the specific species used to treat certain disorders and *N* is the total number of the informants who utilized the species as medicine for treating any given disorder.

#### 2.3.3. Internetwork Analysis (INA)

Internetwork analysis does not focus on the independent characteristics of an individual within the community, but it considers the results of the interrelationship among each individual of a community. Internetwork analysis has been applied within communities to various ethnographical problems, including ethnogenesis [26] and obesity [27–29]; however, prior to this research, the internetwork analysis had yet to be applied to ethnomedicinal knowledge, included with its ethnographical properties in the results.

Our research newly applied this method in order to attain more internetwork information from the treatment of ethnomedicinal practices on liver disorders within communities in Korea. The results of the internetwork analysis of disorders and medicinal species were analyzed using UCINET (Ver. 6.460) and NetDraw (Ver. 2.125) software [30, 31].

## 3. Results and Discussion

### 3.1. Demographic Characteristics of the Region

All 2,069 informants were randomly selected at the community halls, the senior welfare centers, and the traditional markets at 305 sites. Among them, ethnomedicinal practices for liver disorders were recorded by 1,543 informants (362 men, 1,181 women) at 160 sites (Figure 1). The average age of the informants was 75 years old, with a range in age from 36 to 94, with residents living more than 40 years in the study area. The ethnographical characteristics of the communities are summarized in Table 1.

Linguistically, the inland communities represented varying properties between the eastern and western communities, the Jirisan axis (1,915 m). The pronunciation of the two local communities depicts dissimilar intonations, while the languages of the communities on Jeju Island possess numerous dialects different from the inland communities.

In regard to foods, the local communities in the eastern region widely used the seed powder of *Zanthoxylum piperitum* (L.) DC. and the leaves of the *Isodon japonicus* (Burm.) Hara, while local communities in the western region did not consume these foods. Also, the food traditions in communities on Jeju Island are quite diverse from foods of the inland communities in regard to the recipe and ingredients.

The local communities in the east are politically conservative, while local inland communities in the west are more progressive. However, the communities on Jeju Island display extreme exclusiveness because of their historical experiences.

In homes within the inland communities, men usually support their families financially, while women traditionally support their families on Jeju Island.

### 3.2. Analysis of Ethnomedicinal Practices

The kinds of liver disorders treated by ethnomedicinal practices were liver cancer, liver cirrhosis, jaundice, hepatitis, fatigue recovery, hangovers, and liver-related ailments (Table 2). Also, this study area is three times larger than previous research, while the seven types of liver disorders recorded in this study were less than previous research, which classified 14 types of respiratory system diseases, 29 types of digestive system diseases, and 23 types of pain relief treatment [7–9]. We believe that the communities of this study area possess relative health issues related to liver conditions compared to other health concerns.

The 254 ethnomedicinal practices recorded from the communities were classified into 55 families, 85 genera, and 94 species that included plants, animals, and fungi (Table 2). Among these species, plants totaled 150 ethnomedicinal practices based on 31 families, 52 genera, and 57 species while animals included 99 ethnomedicinal practices based on 21 families, 30 genera, and 34 species. Fungi recorded five ethnomedicinal practices based on three families, three genera, and three species. These usage patterns were different from Korean traditional medicine, in which plants are used relatively much more than animals. Research confirms that the communities have focused on direct nutritional supplements from the traditional medicine rather than seek after an actual cure for their liver disorders.

This supposition was confirmed by the fact that liver-related ailments and jaundice require a greater necessity for nutritional supplements than other diseases which are contained within many medicinal species and various ethnomedicinal practices. 

Namely, the number of medicinal species and ethnomedicinal practices for liver-related ailments consisted of 59 species (62.7% of the total species) and 143 ethnomedicinal practices (56.3% of the total practices). Jaundice used 25 species (26.6% of the total species) and 50 ethnomedicinal practices (19.7% of the total practices).

Also, the number of informants who mentioned liver-related ailments and cases of jaundice occupied 58%, which totaled 32% of the whole, respectively (Table 2). As a result, the communities tended to use ethnomedicinal practices to care for their overall health instead of as a cure for a long-term condition.

For plants, 13 used parts were used in practice, while 9 used parts of animals and one used part of fungi were used in treatment. Preparations of the plants consisted of 24 kinds, with 19 preparations for animals and two preparations for fungi (Table 2). The usage recorded is similar to previous research for other diseases [7–9]. 

Among the medicinal species, the most often mentioned plants were *Artemisia capillaris* Thunb., *Taraxacum platycarpum* Dahlst., and *Hovenia dulcis* Thunb. (50.99% mentioned), while the common animals were *Protaetia brevitarsis seulensis* (Kolbe), *Semisulcospira libertina* (Gould), and *Semisulcospira forticosta* (Martens) (6.90% mentioned). The number of mentioned plants focused more on minor species than animal species. Through continued research, these species can certainly be developed into functional foods for particular liver disorders.

### 3.3. Quantitative Analysis

#### 3.3.1. Informant Consensus Factor (ICF)

The informant consensus factor ranges from 0 to 1, where the increasing values indicate a higher rate of informant consensus among the illness category. The category with the highest degree of consensus from the informants was jaundice (0.95), followed by liver-related ailments and fatigue recovery (0.93), hepatitis (0.87), and a hangover (0.86). The lowest degree of consensus was for liver cancer and liver cirrhosis (0.61). These results inform that ethnomedicinal practices have been applied more often to minor health issues related to the liver.

More often, people suffering from serious liver disorders have been treated in the hospital using conventional medicine or Korean traditional medicine. However, ethnomedicinal practices have been used to cure jaundice, liver-related ailments, and fatigue recovery.

#### 3.3.2. Fidelity Level (FL)

The FL is useful for identifying the informants' most preferred species in use for treating certain liver disorders.

This information reveals that the informants had a tendency to rely on one specific species for treating one specific ailment rather than for several different ailments. The FL values in this study varied from 1.0% to 100%. 

Generally, a FL of 100% for a specific species indicates that all of the usereports mentioned the same species for a specific treatment [32]. This study determined 28 species of plants with a FL of 100%, even without considering species that were mentioned above five times (Table 2). Diseases containing a higher number of species assessed to a FL of 100% were liver-related ailments (43 species) and cases of jaundice (16 species).

Special attention was given to important species (*N*, *N*_*p*_) with a FL above 100%, regarding the viewpoint of the number of times mentioned and the consensus level for the specific ailment, like *Taraxacum platycarpum* Dahlst. (192, 192), *Cudrania tricuspidata* (Carr.) Bureau ex Lavallee (43, 43), *Semisulcospira libertina* (Gould) (30, 30), *Capsella bursapastoris* (L.) L. W. Medicus (24, 24), and *Semisulcospira forticosta* (Martens) (10, 10) (Table 2). Through further clinical study, these species possess a much higher potential in being used in the development of new drugs for liver disorders.

#### 3.3.3. Internetwork between Liver Disorders and Medicinal Species

INA has originally analyzed social phenomenon and trends through the internetwork of components [33]. We attempted to analyze the interrelationship between liver disorders and the medicinal species recorded in the communities.

**Figure 2:**
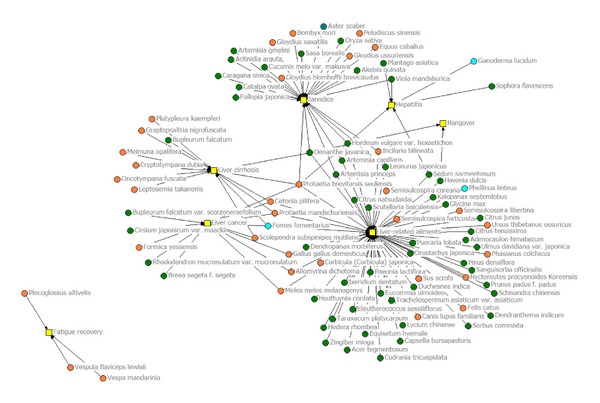
Internetwork analysis between medicinal species and diseases.

Considering Figure 2, the people in the communities used only animals to care for fatigue recovery and liver cirrhosis, except for the use of *Bupleurum falcatum* L. (plant) and *Fomes fomentarius* (L. : Fr.) Fr. (fungus), while using plants and fungi as a cure for hepatitis and hangovers. Also, people used plants, animals, and fungi to treat jaundice, liver cancer, and other liver-related ailments.

*Protaetia brevitarsis seulensis* (Kolbe), used as a medicinal animal, was applied as treatment for five liver disorders, which included liver cancer, liver cirrhosis, jaundice, liver-related ailments, and hepatitis. *Oenanthe javanica* (Blume) DC., used as a medicinal plant, was applied as treatment for the four liver disorders of liver cirrhosis, jaundice, liver-related ailments, and hangovers. *Protaetia mandschuriensis* (Schurhoff) and *Cetonia pilifera* (Motschulsky) were used as medicinal animals and *Fomes fomentarius* (L. : Fr.) Fr. as a medicinal fungus in the treatment of three disorders, which included liver cancer, liver cirrhosis, and liver-related ailments. As further research is conducted, these species will certainly be developed as pharma foods used in treatment of liver disorders.

## 4. Conclusion

This research stands as the first study to record and analyze ethnomedicinal practices used as treatment for liver disorders within the communities in East Asia. After the 1950s, the National Health Care System legally admitted conventional medicine and Korean traditional medicine, which resulted in the near extinction of ethnomedicinal practice in Korea. Also, the fast westernization of local communities in Korea has accelerated the loss of ethnomedicinal practices.

 From this research, recording 254 ethnomedicinal practices, as being used to treat seven liver disorders, was very inspiring. Particularly, the present usage of various bioorganisms displays evidence as to which ethnomedicinal practices are continuously transmitted in the communities. However, this present situation is not sustainable because the communities of these study areas consist of an aging society. It has become necessary for appropriate measures to be taken to conserve these ethnomedicinal practices.

Optimistically, the INA was first applied for the interpretation of ethnomedicinal knowledge of a community, although it has been strictly used until now for social science in the analysis of social trends and phenomena through the interrelationship of specific components.

The results of the INA application in this study provide various interpretations between liver disorders and medicinal species. Our research suggests an internetwork analysis as a new tool for various interpretations to ethnomedicinal knowledge within a local community. Through this study, we are confident that the useful value of INA has been proven and the three dimensional relationships of these components will extend beyond the existing understanding of ethnomedicinal knowledge within local communities around the world.

## Figures and Tables

**Table 1:** Demographic characteristics.

Gender	
Male	362 (23.5%)
Female	1,181 (76.5%)
Age	
30–39	9 (0.6%)
40–49	4 (0.3%)
50–59	73 (4.7%)
60–69	250 (16.2%)
70–79	707 (45.8%)
80–89	460 (29.8%)
90–99	40 (2.6%)
Educational attainment	
Never attended school	1,127 (73.0%)
Attended school for less than 6 years	125 (8.1%)
Attended school for 6 years	133 (8.6%)
Finished middle school	101 (6.5%)
Finished high school	57 (3.7%)

**Table 2:** Information about ethnomedicinal practices recorded from residents in the research area.

Liver disorders	Scientific name	Korean name	Number of mentions	Kind	Used part	Preparation	Application	FL
Fatigue recovery	*Plecoglossus altivelis* Temminck et Schlegel	Euneo	1	Animal	Whole part	Sashimi	Oral	100.0
	*Vespa mandarinia* (Cameron)	Jangsumalbeol	12	Animal	Hive, imago, larva	Dissolution	Oral	100.0
	*Vespula flaviceps lewisii* (Cameron)	Ttangbeol	18	Animal	Hive, imago, larva	Dissolution	Oral	100.0
Hangover	*Hovenia dulcis* Thunb.	Heotgaenamu	4	Plant	Fruit, leaf	Decoction	Oral	3.2
	*Oenanthe javanica* (Blume) DC.	Minari	4	Plant	Aerial part	Maceration	Oral	10.3
Hepatitis	*Ganoderma lucidum* (Leyss.: Fr.) Karst.	Yeongjibeoseot	1	Fungi	Whole part	Decoction	Oral	100.0
	*Plantago asiatica* L.	Jilgyeongi	2	Plant	Leaf	Decoction	Oral	100.0
	*Protaetia brevitarsis seulensis* (Kolbe)	Huinjeombagikkonmuji	10	Animal	Larva	Decoction	Oral	21.7
	*Sedum sarmentosum* Bunge	Dollamul	18	Plant	Whole part	Juice	Oral	22.5
	*Sophora flavescens* Solander ex Aiton	Gosam	7	Plant	Root	Pill	Oral	100.0
	*Viola mandshurica* W. Becker	Jebikkot	2	Plant	Whole part	Decoction	Oral	50.0
Jaundice	*Actinidia arguta* (Siebold & Zucc.) Planch. ex Miq.	Darae	3	Plant	Sap	Raw	Oral	100.0
	*Akebia quinata* (Houtt.) Decne.	Eureumdeonggul	4	Plant	Stem	Decoction	Oral	100.0
	*Artemisia capillaris* Thunb.	Sacheolssuk	344	Plant	Aerial part, leaf, stem, whole part	A sweet drink made from fermented rice, brewing, decoction, dried, grain syrup, infusion, juice, pill, simmer	Oral	75.6
	*Artemisia gmelinii* Weber ex Stechm.	Deowijigi	1	Plant	Whole part	A sweet drink made from fermented rice, brewing, infusion	Oral	100.0
	*Artemisia princeps *Pamp.	Ssuk	10	Plant	Leaf	Rubbing	Topical	83.3
	*Aster scaber* Thunb.	Chamchwi	8	Plant	Leaf	Parboiled	Oral	100.0
	*Bombyx mori* (Linne)	Nuenabang	1	Animal	Larva	Dried, powder, steam	Oral	100.0
	*Caragana sinica* (Buc'hoz) Rehder	Goldamcho	8	Plant	Root	A sweet drink made from fermented rice	Oral	100.0
	*Catalpa ovata* G. Don	Gaeodong	1	Plant	Root	Decoction	Oral	100.0
	*Cucumis melo* var. *makuwa* Makino	Chamoe	19	Plant	Fruit, peduncle	Dried, powder	Topical	100.0
	*Equus caballus* Linn.	Mal	8	Animal	Hide, meat	Infusion, raw	Oral	100.0
	*Fallopia japonica* (Houtt.) RonseDecr.	Hojanggeun	2	Plant	Root	Decoction	Oral	100.0
	*Gloydius blomhoffii brevicaudus *Stejneger	Salmosa	1	Animal	Whole part	Raw	Oral	100.0
	*Gloydius saxatilis* Emelianov	Kkachisalmosa	1	Animal	Whole part	Raw	Oral	100.0
	*Gloydius ussuriensis* Emelianov	Soesalmosa	1	Animal	Whole part	Raw	Oral	100.0
	*Hordeum vulgare* var. *hexastichon* (L.) Asch.	Bori	31	Plant	Malt, seed	A sweet drink made from fermented rice, tea	Oral	66.0
	*Incilaria bilineata* (Benson)	Mindalpaengi	3	Animal	Whole part	Dried, dissolution, maceration, roast	Oral	75.0
	*Leonurus japonicus* Houtt.	Ingmocho	16	Plant	Aerial part	A sweet drink made from fermented rice, infusion, juice, pill, tea	Oral	80.0
	*Oenanthe javanica* (Blume) DC.	Minari	8	Plant	Aerial part, leaf, stem	Seasoned cooked vegetables, juice	Oral	20.5
	*Oryza sativa* L.	Byeo	10	Plant	Seed	A sweet drink made from fermented rice	Oral	100.0
	*Pelodiscus sinensis* Wiegmann	Jara	1	Animal	Whole part	Simmer	Oral	100.0
	*Plantago asiatica* L.	Jilgyeongi	2	Plant	Leaf	Decoction	Oral	100.0
	*Protaetia brevitarsis seulensis* (Kolbe)	Huinjeombagikkonmuji	3	Animal	Whole part	Dried, panbroiled, powder	Oral	6.5
	*Sasa borealis* (Hack.) Makino	Joritdae	3	Plant	Root	Decoction	Oral	100.0
	*Viola mandshurica* W. Becker	Jebikkot	2	Plant	Whole part	Decoction	Oral	50.0
Liver cancer	*Allomyrina dichotoma* (Linne)	Jangsupungdengi	3	Animal	Larva	Dried, infusion, pill, powder, steam	Oral	20.0
	*Breea segeta* (Willd.) Kitam. f. *segeta *	Jobaengi	2	Plant	Root	Decoction	Oral	100.0
	*Bupleurum falcatum* var. *scorzoneraefolium* (Willd.) Ledeb.	Chamsiho	2	Plant	Whole part	Decoction	Oral	100.0
	*Cetonia pilifera* (Motschulsky)	Kkonmuji	5	Animal	Larva, whole part	Dried, infusion, panbroiled, pill, powder, simmer	Oral	38.5
	*Cirsium japonicum* var. *maackii *(Maxim.) Matsum.	Eonggeongkwi	2	Plant	Root	Decoction	Oral	100.0
	*Fomes fomentarius* (L.: Fr.) Fr.	Malgupbeoseot	1	Fungi	Whole part	Infusion	Oral	20.0
	*Formica yessensis* Wheeler	Bulgaemi	1	Animal	Whole part	Decoction, simmer	Oral	100.0
	*Gallus gallus domesticus* Linn.	Dak	1	Animal	Whole part	Decoction, simmer	Oral	11.1
	*Meles meles melanogenys* Allen & Andrew.	Osori	1	Animal	Gall bladder	Dried, mixed in liquor	Oral	50.0
	*Protaetia brevitarsis seulensis* (Kolbe)	Huinjeombagikkonmuji	5	Animal	Larva, whole part	Dried, infusion, panbroiled, powder, pill, simmer	Oral	10.9
	*Protaetia mandschuriensis* (Schurhoff)	Manjujeombagikkonmuji	5	Animal	Larva, whole part	Dried, infusion, panbroiled, powder, pill, simmer	Oral	38.5
	*Rhododendron mucronulatum* Turcz. var. *mucronulatum *	Jindallae	2	Plant	Flower	Fermentation	Oral	100.0
	*Scolopendra subspinipes mutilan *L. Koch	Jine	2	Animal	Whole part	Decoction, simmer	Oral	33.3
Liver cirrhosis	*Bupleurum falcatum* L.	Siho	2	Plant	Root	Decoction	Oral	100.0
	*Cetonia pilifera* (Motschulsky)	Kkonmuji	4	Animal	Larva, whole part	Dried, panbroiled, powder, simmer	Oral	30.8
	*Cryptotympana dubia* (Haupt)	Malmaemi	2	Animal	Larva	Dried, powder, pill	Oral	100.0
	*Fomes fomentarius* (L.: Fr.) Fr.	Malgupbeoseot	1	Fungi	Whole part	Decoction	Oral	20.0
	*Graptopsaltria nigrofuscata *(Motschulsky)	Yujimaemi	3	Animal	Larva	Dried, powder, pill	Oral	100.0
	*Leptosemia takanonis* (Matsumura)	Soyosanmaemi	2	Animal	Larva	Dried, powder, pill	Oral	100.0
	*Meimuna opalifera* (Walker)	Aemaemi	2	Animal	Larva	Dried, powder, pill	Oral	100.0
	*Oenanthe javanica* (Blume) DC.	Minari	2	Plant	Aerial part	Juice	Oral	5.1
	*Oncotympana fuscata* (Distant)	Chammaemi	2	Animal	Larva	Dried, powder, pill	Oral	100.0
	*Platypleura kaempferi* (Fabricius)	Teolmaemi	2	Animal	Larva	Dried, powder, pill	Oral	100.0
	*Protaetia brevitarsis seulensis* (Kolbe)	Huinjeombagikkonmuji	4	Animal	Larva, whole part	Dried, panbroiled, powder, simmer	Oral	8.7
	*Protaetia mandschuriensis* (Schurhoff)	Manjujeombagikkonmuji	4	Animal	Larva, whole part	Dried, panbroiled, powder, simmer	Oral	30.8
	*Scolopendra subspinipes mutilans *L. Koch	Jine	2	Animal	Whole part	Dried, powder, pill	Oral	33.3
Liver-related ailments	*Acer tegmentosum* Maxim.	Sangyeoreumnamu	3	Plant	Stem	Decoction, tea	Oral	100.0
	*Adenocaulon himalaicum* Edgew.	Myeolgachi	2	Plant	Whole part	Juice	Oral	100.0
	*Allomyrina dichotoma* (Linne)	Jangsupungdengi	12	Animal	Larva, whole part	Dissolution, dried, extraction, infusion, panbroiled, powder, roast, simmer	Oral	80.0
	*Artemisia capillaris* Thunb.	Sacheolssuk	111	Plant	Aerial part, leaf, whole part	A sweet drink made from fermented rice, decoction, grain syrup, infusion, juice, pill, simmer	Oral	24.4
	*Artemisia princeps* Pamp.	Ssuk	2	Plant	Whole part	Juice	Oral	16.7
	*Canis lupus familiaris *Linn.	Gae	1	Animal	Whole part	Infusion	Oral	100.0
	*Capsella bursapastoris* (L.) L.W.Medicus	Naengi	24	Plant	Whole part	Juice, seasoned cooked vegetables, soup	Oral	100.0
	*Cetonia pilifera* (Motschulsky)	Kkonmuji	4	Animal	Larva	Dried, panbroiled, powder, simmer	Oral	30.8
	*Citrus junos* Siebold ex Tanaka	Yujanamu	6	Plant	Fruit	Decoction	Oral	100.0
	*Citrus natsudaidai* Hayata	Hagyul	6	Plant	Fruit	Maceration	Oral	100.0
	*Citrus tenuissima* Tanaka.	Dangyujanamu	10	Plant	Fruit	Decoction	Oral	100.0
	*Corbicula (Corbicula) japonica* Prime	Ilbonjaecheop	8	Animal	Whole part	Simmer	Oral	100.0
	*Cudrania tricuspidata* (Carr.) Bureau ex Lavallee	Kkujippongnamu	43	Plant	Bark, stem	A sweet drink made from fermented rice, decoction, infusion	Oral	100.0
	*Dendranthema indicum* (L.) DesMoul.	Gamguk	3	Plant	Flower	Decoction	Oral	100.0
	*Dendropanax morbiferus* H. Lev.	Hwangchillamu	4	Plant	Leaf, stem	Decoction	Oral	100.0
	*Duchesnea indica* (Andr.) Focke	Baemttalgi	2	Plant	Fruit	Decoction	Oral	100.0
	*Eleutherococcus sessiliflorus* (Rupr. & Maxim.) S. Y. Hu	Ogalpinamu	19	Plant	Fruit, stem	Extraction, infusion	Oral	100.0
	*Equisetum hyemale* L.	Soksae	2	Plant	Stem	Decoction	Oral	100.0
	*Eucommia ulmoides* Oliv.	Duchung	2	Plant	Stem	Decoction	Oral	100.0
	*Felis catus* Linn.	Goyangi	1	Animal	Whole part	Simmer	Oral	100.0
	*Fomes fomentarius *(L.: Fr.) Fr.	Malgupbeoseot	3	Fungi	Whole part	Decoction	Oral	60.0
	*Gallus gallus domesticus* Linn.	Dak	8	Animal	Whole part	Infusion	Oral	88.9
	*Glycine max* (L.) Merr.	Kong	2	Plant	Seed	Steeped in vinegar	Oral	100.0
	*Hedera rhombea* (Miq.) Bean	Songak	2	Plant	Fruit	Decoction	Oral	100.0
	*Hordeum vulgare* var. *hexastichon* (L.) Asch.	Bori	16	Plant	Malt	A sweet drink made from fermented rice, pill	Oral	34.0
	*Houttuynia cordata* Thunb.	Yangmomil	5	Plant	Whole part	Decoction, infusion	Oral	100.0
	*Hovenia dulcis* Thunb.	Heotgaenamu	121	Plant	Fruit, leaf, stem	Decoction, infusion, tea	Oral	96.8
	*Incilaria bilineata* (Benson)	Mindalpaengi	1	Animal	Whole part	Dissolution, dried, powder	Oral	25.0
	*Ixeridium dentatum* (Thunb. ex Mori) Tzvelev	Sseumbagwi	5	Plant	Whole part, young leaf	Juice	Oral	100.0
	*Kalopanax septemlobus* (Thunb.) Koidz.	Eumnamu	9	Plant	Stem	Decoction, infusion	Oral	100.0
	*Leonurus japonicus* Houtt.	Ingmocho	4	Plant	Aerial part	Infusion, pill	Oral	20.0
	*Lycium chinense* Mill.	Gugijanamu	8	Plant	Fruit	Infusion	Oral	100.0
	*Meles meles melanogenys* Allen & Andrew.	Osori	1	Animal	Gall bladder	Brewing	Oral	50.0
	*Nyctereutes procyonoides Koreensis* Mori.	Neoguri	2	Animal	Gall bladder	Brewing	Oral	100.0
	*Oenanthe javanica* (Blume) DC.	Minari	25	Plant	Aerial part, stem, whole part	Juice	Oral	64.1
	*Orostachys japonica* (Maxim.) A.Berger	Bawisol	2	Plant	Whole part	Decoction, juice	Oral	100.0
	*Paeonia lactiflora* Pall.	Jagyak	2	Plant	Root	Decoction, roast	Oral	100.0
	*Phasianus colchicus* Linn.	Kkwong	1	Animal	Whole part	Soup	Oral	100.0
	*Phellinus linteus* (Berk. et Curt.) Teng	Mokjiljinheukbeoseot	3	Fungi	Whole part	Decoction	Oral	100.0
	*Pinus densiflora* Siebold & Zucc.	Sonamu	4	Plant	Leaf	Dried, powder, steam	Oral	100.0
	*Protaetia brevitarsis seulensis* (Kolbe)	Huinjeombagikkonmuji	24	Animal	Larva, whole part	Decoction, dissolution, dried, extraction, panbroiled, powder, simmer	Oral	52.2
	*Protaetia mandschuriensis* (Schurhoff)	Manjujeombagikkonmuji	4	Animal	Larva	Dried, panbroiled, powder, simmer	Oral	30.8
	*Prunus padus* L. for. *padus *	Gwirungnamu	2	Plant	Stem	Decoction	Oral	100.0
	*Pueraria lobata* (Willd.) Ohwi	Chik	1	Plant	Root	Decoction	Oral	100.0
	*Sanguisorba officinalis* L.	Oipul	2	Plant	Root	Decoction	Oral	100.0
	*Schisandra chinensis* (Turcz.) Baill.	Omija	6	Plant	Fruit, root, stem	Brewing	Oral	100.0
	*Scolopendra subspinipes mutilans* L. Koch	Jine	2	Animal	Whole part	Dissolution, dried, powder	Oral	33.3
	*Scutellaria baicalensis* Georgi	Hwanggeum	1	Plant	Root	Decoction	Oral	100.0
	*Sedum sarmentosum* Bunge	Dollamul	62	Plant	Aerial part	Juice, maceration, powder, raw, seasoned cooked vegetables, watery plain kimchi	Oral	77.5
	*Semisulcospira coreana* (Martens)	Chamdaseulgi	11	Animal	Whole part	Clear soup with flour dumplings, infusion, juice, simmer	Oral	100.0
	*Semisulcospira forticosta* (Martens)	Jureumdaseulgi	23	Animal	Whole part	Clear soup with flour dumplings, infusion, juice, simmer	Oral	100.0
	*Semisulcospira libertina* (Gould)	Daseulgi	30	Animal	Body, whole part	Clear soup with flour dumplings, infusion, juice, soup, simmer	Oral	100.0
	*Sorbus commixta* Hedl.	Magamok	10	Plant	Fruit, leaf, stem	Decoction, tea	Oral	100.0
	*Sus scrofa* Linn.	Metdwaeji	6	Animal	Gall bladder	Dissolution, juice	Oral	100.0
	*Taraxacum platycarpum* Dahlst.	Mindeulle	192	Plant	Aerial part, leaf, whole part	A sweet drink made from fermented rice, decoction, dried, extraction, infusion, juice, kimchi, raw, seasoned cooked vegetables, simmer, tea	Oral	100.0
	*Trachelospermum asiaticum* (Siebold & Zucc.) Nakai var. *asiaticum *	Masakjul	4	Plant	Leaf, stem	Decoction	Oral	100.0
	*Ulmus davidiana* var. *japonica* (Rehder) Nakai	Neureumnamu	8	Plant	Bark	Decoction	Oral	100.0
	*Ursus thibetanus ussuricus* Heude	Bandalgaseumgom	1	Animal	Flesh	Infusion	Oral	100.0
	*Zingiber mioga* (Thunb.) Roscoe	Yangha	2	Plant	Whole part	Juice	Oral	100.0
